# The prognostic value of B7-H6 in esophageal squamous cell carcinoma

**DOI:** 10.1038/s41598-019-54731-9

**Published:** 2019-12-02

**Authors:** Huan Zhou, Jun Dong, Liyi Guo, Xicheng Wang, Kailin Wang, Xiuyu Cai, Shu Yang

**Affiliations:** 1 Department of Oncology, The First Affiliated Hospital of Guangdong Pharmaceutical University, Guangzhou, Guangdong China; 2 Department of VIP Region, Sun Yat-Sen University Cancer Center, Guangzhou, Guangdong China; 3 Department of Radiotherapy, Huiyang Hospital Affiliated Southern Medical University, Huizhou, Guangdong China

**Keywords:** Oesophageal cancer, Tumour biomarkers

## Abstract

B7-H6, a member of the B7 family molecules, participates in the clearance of tumor cells by binding to NKp30 on NK cells. B7-H6 expression level in esophageal squamous cell carcinoma (ESCC) and the clinical value remain unknown. The goal of this study was to determine the expression of B7-H6 in ESCC and further explore its clinical significance. We retrospectively collected the clinical data of 145 patients diagnosed with ESCC between January 2007 and December 2008. The expression of B7-H6 of the pathological tissue samples was detected by immunohistochemistry. The chi-square (χ^2^) test was used to analyse the relationships of B7-H6 and clinicopathological characteristics. Survival and hazard functions were estimated using the Kaplan-Meier method, and survival between groups was compared using the two-sided log-rank test. The Cox proportional hazards regression model was used to adjust for the risk factors related to overall survival (OS). 133/145 (91.72%) of the ESCC tissue samples exhibited B7-H6 expression. The expression level of B7-H6 was correlated with T stage (*P* = 0.036) and lymphatic metastasis status (*P* = 0.044). High B7-H6 expression (*P* = 0.003) was associated with a significantly worse OS than low B7-H6 expression. Multivariate Cox proportional hazards regression analysis demonstrated that tumour size (*P* = 0.021), B7-H6 expression (*P* = 0.025) and lymphatic metastasis status (*P* = 0.049) were independent prognostic factors of OS for ESCC. Collectively, our findings suggest that B7-H6 is widely expressed in ESCC samples. And B7-H6 may represent a predictor of poor prognosis for ESCC.

## Introduction

Esophageal cancer is one of the most common malignant tumours in the world, ranking as the 6th most common cause of cancer-related death and the 8th most common cancer in the world, with an increasing incidence^1^. Esophageal squamous cell carcinoma (ESCC) accounts for about 90% of esophageal cancer cases, which always in advanced stage when first diagnosed, with a low 5-year overall survival (OS) rate of about15~25%^1^. Recently, with the rapid development of surgery, radiotherapy, chemotherapy, biological therapy and other comprehensive treatment methods, the prognosis of ESCC is still poor. Therefore, in-depth studies of the molecular mechanism underlying the occurrence and development of ESCC and the search for new molecular targets for diagnosis and prognostic monitoring have important clinical application value.

Tumour immune escape is an important molecular mechanism in the processes of tumourigenesis, invasion and metastasis. Tumour cells are usually unable to provide effective antigen signals, or the body has a defective immune response and is immunosuppressed, Further, they escape the surveillance and monitoring of the immune effector cells^2^. In this process, costimulatory molecules and their regulatory networks play an important role. Costimulatory molecules are mainly divided into two superfamilies, B7/CD28 and TNF/TNFR^3^. Recently, new discovered members of the B7 family of costimulatory molecules, such as B7-H1, B7-H3, B7-H4 and B7-H6, are widely expressed in many human tumour tissue types and can participate in the negative regulation of the T/natural killer (NK) cell-mediated antitumour immune response, thus attracting wide attention^3,4^. Studies have revealed that the expression of PD-L1 (B7-H1) and B7-H3 in esophageal cancer were associated with prognosis^5–7^. Ling Wang *et al*. reported B7-H3 and B7-H4 were widely expressed in ESCC. The high expression level of B7-H3 and B7-H4 were related to TNM stages and lymph node metastasis. Patients with both high levels of B7-H3 and B7-H4 had the poorest prognosis^8^. Additionally, Lijie Chen *et al*. demonstrated that B7-H4 expression was associated with ESCC progression and survival by reducing tumor immunosurveillance^9^.

B7-H6 is a recently discovered member in the B7 family^10,11^. It is a type I transmembrane protein that shows similar structure to B7-H1 and B7-H3 proteins^12^. The extracelluar region of B7-H6 consists of an IgV-like domain and an IgC-like domain. Gordon Joyce *et al*. have verified that the extracellular region of NKp30 interacts with the extracellular region of B7-H6 by directly and selectively binding, which used residue mutation strategy^13^. Studies have shown that B7-H6 can be activated by binding with the activation receptor NKp30 on the surface of NK cells and that B7-H6 promotes TNF- and IFN-mediated killing of tumour cells by NK cells, which is one of the important mechanisms of NK cell-mediated antitumour immunity^12^. B7-H6 expression in mRNA level was found in human primary lymphoma, leukemia, ovarian cancer, brain tumour, breast cancers, renal cell carcinoma, and various sarcomas^11^. The B7-H6 expression level was significantly upregulated in tumour tissue samples compared with the normal tissue, which was closely related to the clinicopathological characteristics and prognosis of patients^14–17^. However, until now, the clinical significance of B7-H6 expression in ESCC has not been reported. In this study, we investigated B7-H6 expression in ESCC tissue samples and explored the clinical implications.

## Results

### Study population

Patient characteristics were shown in Table 1. The median age of the study group was 60 years (range from 34 to 88 years). 82 cases (56.6%) patients were diagnosed with grade I-II and 63 cases (43.4%) were grade III- IV, according to TNM staging. Of the 145 patients examined, 79 (54.5%) died before the last follow-up evaluation period.

**Table 1:** Clinical characteristics of 145 patients.

Characteristic	Number (%)
Age(Years)	
Median	60
Range	34–88
<60	71(49.0)
≥60	74(51.0)
Gender	
Male	107(73.8)
Female	38(26.2)
Tumour size (cm)	
<3.0	69(47.6)
≥3.0	75(51.7)
Tumour location	
Upper third	14(9.7)
Middle third	90(62.1)
Lower third	41(28.3)
Differentiation grade	
Low	29(20.0)
Middle	60(41.4)
High	32(22.1)
Low-middle	13(9.0)
Middle-high	11(7.6)
T stage	
T1	13(9.0)
T2	23(15.9)
T3	96(66.2)
T4	13(9.0)
Lymphatic metastasis	
Yes	64(44.1)
No	81(55.9)
TNM stages	
I + II	82(56.6)
III + IV	63(43.4)
Death	
Yes	79(54.5)
No	66(45.5)

### B7-H6 expression in esophageal tissue

**Figure 1:**
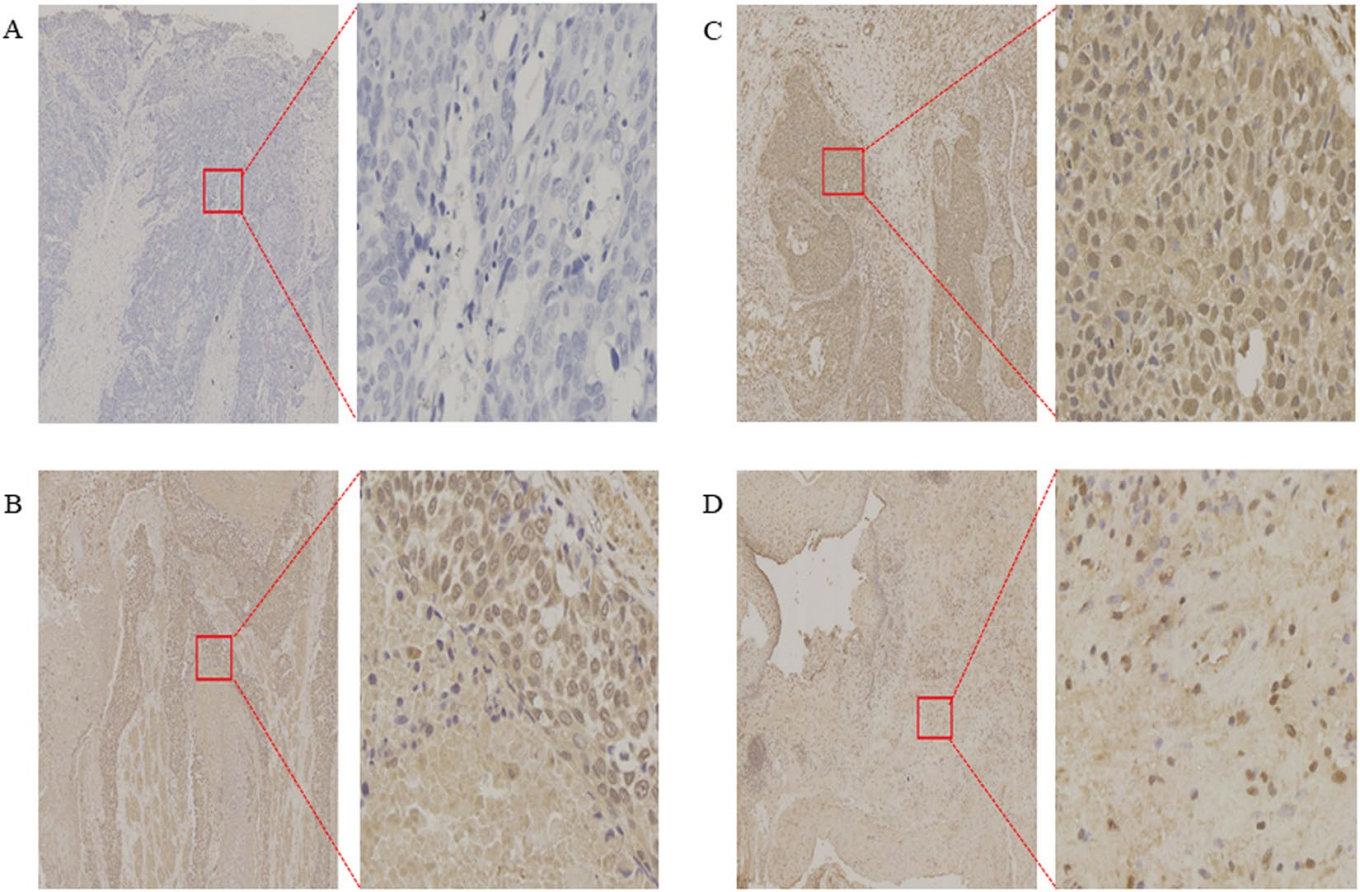
Immunohistochemical staining (magnification: left, 5×; right, 100×). Results: B7-H6 expression (brown) in ESCC tissue samples and adjacent normal tissue samples. Negative B7-H6 expression (**A**); Low B7-H6 expression (**B**); High B7-H6 expression in ESCC tissue samples(**C**). Low B7-H6 expression in adjacent normal tissue samples(**D**).

To detect the B7-H6 expression level in ESCC tissue, immunohistochemical analysis was used (Fig. 1). The approach showed that B7-H6 was present in 133/145 (91.72%) of the samples, which localized in the cytoplasm, while weak B7-H6 staining was found in normal esophageal tissue. The median value of the B7-H6 staining H-score was 40 (0–180).

### The clinical significance of B7-H6 expression

The clinicopathological characteristics and B7-H6 expression of all patients were presented in Table 2. All patients were divided into two major subgroups according to the median B7-H6 intensity of staining of 40. This stratification revealed that B7-H6 expression was significantly correlated with T stage (*P* = 0.036) and lymphatic metastasis status (*P* = 0.044); however, B7-H6 expression did not correlate with other risk factors, including gender, age, tumour size, tumour location, differentiation grade, TNM stages, local recurrence status and metastasis status (*P* > 0.05).

**Table 2:** The clinical significance of B7-H6 expression.

Clinical parameters	Cases	B7-H6 expression level			P-value
		H-score < 40	H-score ≥ 40	χ^2^	
Gender				0.096	0.756
Male	107	51	56		
Female	38	17	21		
Age (Years)				0.055	0.815
<60	71	34	37		
≥60	74	34	40		
Tumour size,cm				0.652	0.419
<3.0	69	35	34		
≥3.0	75	33	42		
Tumour location				0.66	0.719
Upper third	14	8	6		
Middle third	90	41	49		
Lower third	41	19	22		
Differentiation grade				1.389	0.846
Low	29	14	15		
Middle	60	30	30		
High	32	13	19		
Low-middle	13	5	8		
Middle-high	11	6	5		
T stage				8.526	0.036^a^
T1	13	11	2		
T2	23	9	14		
T3	96	43	53		
T4	13	5	8		
Lymphatic metastasis				4.062	0.044^a^
Yes	64	24	40		
No	81	44	37		
TNM stages				0.73	0.393
I + II	82	41	41		
III + IV	63	27	36		
Local recurrence or metastasis				2.794	0.095
Yes	30	10	20		
No	115	58	57		

Note: ^a^*P* < 0.05.

### Survival outcomes

**Figure 2:**
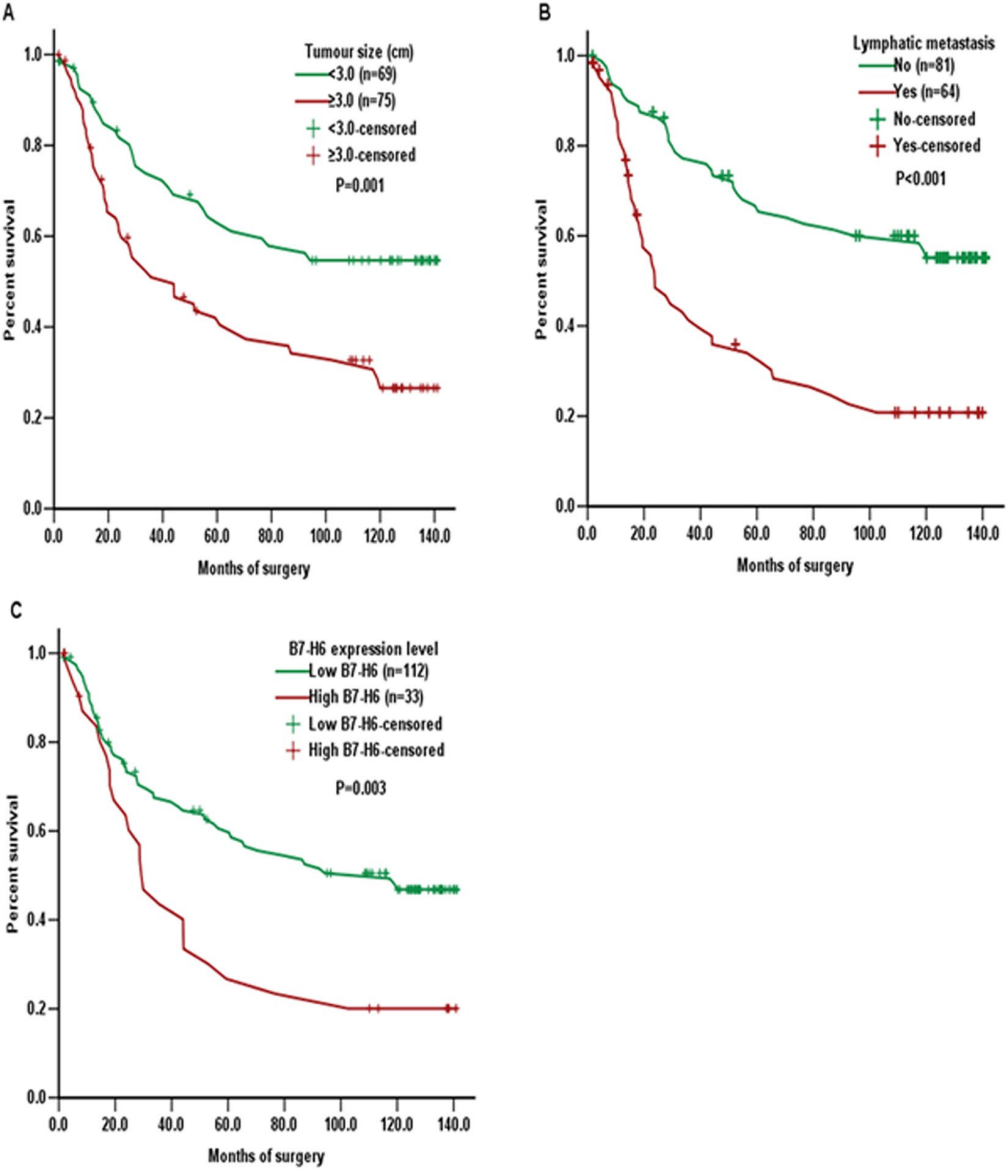
Kaplan-Meier survival analysis of the associations between overall survival and tumour size (**A**, *P* = 0.001), lymphatic metastasis status (**B**, *P* < 0.001) or B7-H6 expression (**C**, *P* = 0.003) in ESCC patients.

With H-score = 90 as the cut-off value, the 145 patients were divided into two subgroups: low B7-H6 expression group (H-score ≤ 90) and high B7-H6 expression group (H-score > 90). The survival analysis using the log-rank test demonstrated that the patients with tumour size ≥ 3.0 cm (*P* = 0.001; Fig. 2A), with lymphatic metastasis (*P* < 0.001; Fig. 2B) and high B7-H6 expression (*P* = 0.003; Fig. 2C) expressed significantly worse survival, respectively. Univariate Cox regression analysis demonstrated that the clinical parameters tumour size, T stage, lymphatic metastasis, TNM stages and B7-H6 expression were significantly associated with survival. Multi-factors Cox regression analyses showed that tumour size (HR: 1.749, 95% CI: 1.089–2.807; *P* = 0.021), lymphatic metastasis status (HR: 2.157, 95% CI: 1.002–4.644; *P* = 0.049) and B7-H6 expression (HR: 1.751, 95% CI: 1.071–2.861; *P* = 0.025) were independent prognostic factors in ESCC, as shown in Table 3.

**Table 3:** Cox regression model analysis of prognosis in esophageal squamous cell carcinoma.

	Univariable		Multivariable	
	HR (95% CI)	P value	HR (95% CI)	P value
Age, years (<60 vs ≥60)	1.043 (0.670–1.621)	0.853		
Gender (male vs female)	0.814 (0.503–1.315)	0.400		
**Tumour location**				
Upper third vs Lower third Middle third vs Lower third	1.192(0.528–2.693) 0.891(0.535–1.484)	0.673 0.658		
Tumour size, cm (<3.0 vs ≥3.0)	2.095(1.323–3.316)	0.002^a^	1.749(1.089–2.807)	0.021^a^
**T stage**				
T1 vs T4	0.161 (0.044–0.587)	0.006^a^	0.400(0.092–1.745)	0.223
T2 vs T4	0.400 (0.169–0.948)	0.037^a^	0.507(0.140–1.842)	0.302
T3 vs T4	0.567(0.288–1.118)	0.101	0.580(0.258–1.305)	0.188
Lymphatic metastasis (yes vs no)	2.914(1.856–4.577)	<0.001^a^	2.157(1.002–4.644)	0.049^a^
TNM stages (I-II vs III-IV)	2.912 (1.849–4.586)	<0.001^a^	1.186 (0.474–2.969)	0.715
B7-H6 expression level (low vs high)	2.053(1.266–3.329)	0.004^a^	1.751(1.071–2.861)	0.025^a^

Note: ^a^P < 0.05.

Abbreviations: HR = hazard ratio; CI = confidence interval.

## Discussion

The superfamily member B7/CD28 has been shown to play an important role in the immune response, and they are seen as effective markers in cancer diagnosis and treatment^3,18^. B7-H6, the new member of the B7 family, interacted with NK cell surface receptor NKp30 and played an obvious role in NK cell-mediated immune responses^11^. NK cells were important immune cells in the body. It was a core cell of the natural immune system and can kill tumour cells. NK-cells activation was regulated by some activation receptors or inhibition receptors on the cell surface^10^. The major activating receptors included NKG2D and the natural cytotoxicity receptors (NCRs) such as NKp46, NKp30, and NKp44^19^. NKp30 can promote NK cells to recognize and kill tumor cells, either alone or together with other stimulation receptors^20–22^. The HLA–B-associated transcript 3 (BAT3) and the pp65 proteins have been revealed to bind NKp30, but they don’t bind to ligands on the surface of tumour cell because pp65 was a cytomegalovirus tegument protein^23^ and BAT3 was a nuclear protein released after heat-shock treatment^24^. B7-H6 is a potent ligand for NKp30, and it doesn’t bind any other CD28 family members nor other NCRs^12^. B7-H6 expressed on tumour cells contacted NKp30 in a unique way that is the complementarity-determining region (CDR)-like loops of its V-like domain^25^. NK cells eliminate B7-H6-expressing tumour cells either directly via cytotoxicity or indirectly by cytokine secretion^11^. Eva Schlecke *et al*. illustrated that tumour cells impeded NK-mediated recognition by metalloprotease-mediated shedding of B7-H6^26^. Soluble B7-H6 generated by ectodomain shedding is another form of B7-H6^11^. Soluble B7-H6 had the ability to block the connection between anti-NKp30 mAbs and NKp30, thus inhibiting NKp30-mediated NK cell triggering^26,27^. Taken together, these data on B7-H6/ NKp30 interaction provided a theoretical basis for the development of novel cancer treatments.

In recent years, immune checkpoint inhibitors that block cytotoxic T-lymphocyte antigen 4 (CTLA-4) and programmed cell death protein 1 (PD-1) have led to significant improvements in prognosis and have brought tumour immunotherapy into a new era^28,29^. Several clinical studies in esophageal cancer using PD-1 inhibitors, such as nivolumab or pembrolizumab, are in progress with recent promising results^30–32^. However, the relationship between PD-1/PD-L1 expression in esophageal cancer tissue and prognosis remains controversial. To date, no good biomarker has been found to guide treatment and prognosis. Abnormal expression of B7-H6 has been found in many cancers, revealing that B7-H6 expressed important clinical significance. Our study is the first to explore the connection between prognosis and clinical implications of B7-H6 expression in ESCC tissue. The results of IHC staining suggested that B7-H6 expression was present in most ESCC tissue samples, which was consistent with the findings of other studies^14,17,33^. We also found that the expression level of B7-H6 correlated with T stage and lymphatic metastasis status, which suggested that the expression of B7-H6 may be a marker to identify T stage and lymphatic metastasis status of ESCC. The result is similar to the findings of studies in gastric cancer, ovarian cancer, non-small cell lung cancer, astrocytoma, breast cancer and other cancers^14,15,17,33–35^. In addition, Cox regression model analysis and log-rank test demonstrated that the expression level of B7-H6 was an independent prognostic factor for ESCC. Patients with high B7-H6 expression had significantly worse survival than those with low B7-H6 expression, suggesting that high B7-H6 expression is a predictor of poor prognosis. This result is similar to data from other researchers^15,34,35^. Thus, B7-H6 might be a meaningful biomarker for predicting the OS of ESCC patients, and also serve as an independent prognostic index. In the past three years, some studies about the knockdown of B7-H6 expression in tumours have been carried out^16,36,37^ and implied that B7-H6 might be a meaningful target for cancer therapy. Therefore, it is believed that further studies on B7-H6 expression at the gene level and the knockdown of B7-H6 expression may also have certain clinical value in determining the prognosis of ESCC patients.

Still, our study has some limitations. Firstly, its retrospective nature, potential selection bias, and confounding bias, were unavoidable. Secondly, all tumour samples were all from patients of China, which may differ from other ethnics and region. Thirdly, it may be more meaningful to detect B7-H6 expression in the protein and gene level using western bolt, enzyme-linked immunosorbent assay (ELISA) or gene chip. And further validate the phenotype change via altering the expression of B7-H6 in ESCC is meaningful.

## Material and Methods

### Patient characteristics

We retrospectively collected clinical data of 145 patients with ESCC between January 2007 and December 2008 in Sun Yat-sen University Cancer Center (SYSUCC). These patients had all undergone surgery, pathologically diagnosed, and had not received chemotherapy or radiotherapy. Pathological tissue samples from the 145 patients were collected to detect the expression of B7-H6 by immunohistochemistry. In addition, 7 non-malignant esophageal tissue samples were collected and used as controls. The clinicopathological data of all patients were available and were used for statistical analysis. The study was approved by SYSUCC Ethics Committee, and informed consent was provided by all patients based on the Declaration of Helsinki.

### Antibodies and major regents

Anti-B7-H6 antibody (ab121794) was purchased from Abcam (Cambridge, MA, USA; dilution 1/100), and horseradish peroxidase (HRP) secondary antibodies was purchased from Dako (Glostrup, Denmark). A DAB color kit was purchased from Beijing Zhongshan Jinqiao Biotechnology Co., Ltd. (Beijing, China; Cat. No. zli-9017).

### Immunohistochemistry

Paraffin-embedded tissue samples were cut into 5 μm sections and roasted for 30 minutes in a 60 °C constant-temperature box. The sections were dewaxed with xylene and rehydrated with different concentrations of ethanol. Blocking endogenous peroxidase activity with 3% hydrogen peroxide solution for 10 minutes, extracting antigen in sodium citrate buffer (0.01 mmol/L, pH 6.0) at 100 °C for 20 minutes. After being soaked in distilled water for 10 minutes, the sections were incubated with 10% foetal bovine serum to block nonspecific binding. Next, the sections were incubated with rabbit anti-human B7-H6 polyclonal antibody at 4 °C overnight, and then incubated with the HRP-conjugated goat anti-mouse/rabbit secondary antibody at room temperature for 60 minutes. The excess secondary antibody was removed by washing with TBS and developed with DAB colorant; the sections were stained with haematoxylin, dehydrated with an alcohol gradient, dried and sealed with neutral resin.

### Evaluation of immunohistochemical (IHC) staining

The ESCC tissue samples were analyzed by two independent senior pathologists who did not know the clinical and pathological information of the patients. The B7-H6 immunohistochemical staining results were analyzed according to a previously described method^9,38^: H-score = (% non-stained tumour cells × 0) + (% weakly stained tumour cells × 1) + (% moderately stained tumour cells × 2) + (% strongly stained tumour cells × 3). The staining intensity was divided into four grades: “0” (non-stained), “1” (weakly stained), “2” (moderately stained), or “3” (strongly stained). The H-scores ranged from 0 to 300. The average results of two pathologists were taken for statistical analysis.

### Statistical analyses

Overall survival (OS) was used to assess prognostic indicator. It is the time from surgery to patient death or last follow-up. The final follow-up date was December 24, 2018. The B7-H6 high and low expression groups were determined by receiver operating characteristic (ROC) curve analysis. The correlations between the B7-H6 expression level and different clinical parameters were analysed by the chi-square (χ^2^) test. Survival date were analysed using the Kaplan–Meier method. Cox regression model analysis were used to explore the effects of clinical variables and B7-H6 on survival. Statistical significance was defined at *P* < 0.05. All date was analysed by SPSS software (version 13.0; IBM Corp., Armonk, NY, USA).

## Conclusion

Our present study indicated that B7-H6 was widely expressed in ESCC tissues and can serve as an independent prognostic marker for ESCC.

## Data Availability

The data supporting the conclusions is available in the repository [the Research Data Deposit public platform], [RDDA2019001075 in http://www.researchdata.org.cn].
